# Humoral immune response to repeated lumpy skin disease virus vaccination and performance of serological tests

**DOI:** 10.1186/s12917-019-1831-y

**Published:** 2019-03-06

**Authors:** Milovan Milovanović, Klaas Dietze, Vesna Milićević, Sonja Radojičić, Miroslav Valčić, Tom Moritz, Bernd Hoffmann

**Affiliations:** 1 Department of Infectious Diseases of Animals and Diseases of Bees, Faculty of Veterinary Medicine, Blvd. Oslobodjenja 18, Belgrade, 11000 Serbia; 2 Friedrich-Loeffler-Institut, Südufer 10, D-17493 Greifswald-Insel Riems, Germany; 3 Virology Department, Institute of Veterinary Medicine of Serbia, Vojvode Toze 14, Belgrade, 11000 Serbia; 4 Present address: Physiolution GmbH, Walther-Rathenau-Straße 49a, D-17489 Greifswald, Germany

**Keywords:** LSD, LSDV, Humoral immunity, Passive antibody transfer, Secondary response, ELISA

## Abstract

**Background:**

In the presented study we investigated the development of the humoral immune response against LSDV during the process of re-vaccination of cattle over a time span of 5 months. In addition, the performance of different serological techniques for antibody detection against LSDV was compared. For sample collection, an area without previous LSD outbreak reports in Serbia was selected. Seventy-nine cattle from twenty farms vaccinated in 2016 and re-vaccinated in 2017 were included in the study. Two farms from the same area with good calving management were selected for investigation of passive LSDV antibody transfer from vaccinated mothers to new-borne calves.

**Results:**

All investigated cattle were healthy on the day of vaccination and during the whole study. Swelling at the injection site or other side effects of vaccination did not occur after re-vaccination in the study.

Detection of LSD-specific antibodies was performed with the standard serological methods VNT and IFAT as well as a commercially available Capripox double antigen multi-species-ELISA. Capripoxvirus-specific antibodies were detected 46 to 47 weeks after vaccination in 2016, with VNT in 35.06% and with IFAT and ELISA in 33.77%. A secondary response was observed in all three tests 1 month after re-vaccination with a significant increase in seropositive animals compared to the results before re-vaccination. With all applied serological methods, the number of animals testing positive was significantly higher at 1 and 5 months post re-vaccination than before re-vaccination. No significant statistical difference (*p* > 0.05) was observed between the results of all three tests used. The sensitivity and specificity of ELISA was estimated to be Se_ELISA_ 91% and Sp_ELISA_ 87% calculated by the results of VNT and Se_ELISA_ 88% and Sp_ELISA_ 76% calculated by the results of IFAT. Passive antibody transfer from vaccinated mothers to new-born calves was investigated at 14 days after birth. Discrepancies for the detection of LSDV specific antibodies between cows and newborn calves at the age of 14 days were observed in VNT and IFAT, but not in ELISA.

**Conclusion:**

Of all tests used the commercially available ELISA shows to be the most useful for high throughput analysis compared to VNT or IFAT.

## Background

Lumpy skin disease (LSD) is a viral disease of cattle caused by lumpy skin disease virus (LSDV) and is included in the OIE list of notifiable animal diseases [1]. LSDV belongs to the genus *Capripoxvirus* of the family *Poxviridae* together with sheep pox (SPP) and goat pox (GTP) virus [2]. It is widely accepted that transmission of LSDV mainly takes place mechanically by blood-feeding arthropods such as mosquitoes (*Aedes aegypti*), stable flies (*Stomoxys calcitrans*) and ticks (*Amblyomma hebraeum* and *Rhipicephalus appendiculatus*) feeding on infected animals [3–6]. The disease can manifest in different forms ranging from acute to in-apparent, characterized by fever, lymphadenitis, skin nodules, lesions of the ocular, nasal and oral mucous membranes, and can in severe forms sometimes lead to death [7]. Development of nodules of different size starts after onset of fever, the number may range from a few nodules to the generalized form covering the entire body [8].

Over the past decades, LSD has spread from sub-Saharan Africa, where it is considered endemic in many countries, to the Middle East triggering concern of a further spread into Europe and Asia. In 2013, the first LSD outbreak was reported in Turkey where the disease subsequently became endemic. A further spread from Turkey into Greece was seen in 2015, followed by outbreaks in Bulgaria, the Former Yugoslav Republic of Macedonia, Serbia, Albania and Kazakhstan in 2016 [9]. On June 7, 2016 a first LSD outbreak was officially confirmed in Serbia, in the municipality of Bujanovac, Ljiljance village, near the border to the Former Yugoslav Republic of Macedonia [10].

For successful LSD control, vaccination of all susceptible animals is considered to be the main pillar, supported by other control measures such as stamping out, animal movement restrictions and vector control. For vaccination of cattle against LSD, live attenuated capripoxvirus vaccine strains were used, including the homologous LSDV Neethling strain and KSGP O-240 previously described as Kenyan sheep pox and goat pox virus, or the heterologous RM65 SPP, Romanian SPP and Gorgan GTP virus strains [11–14].

Facing the risk of a spread of LSD throughout the country, disease control measures including mass vaccination were applied in Serbia. In 2016, all susceptible animals were vaccinated using a LSDV Neethling vaccine (Onderstepoort Biological Products, South Africa) after dividing the country into three zones. Vaccination in zone one (infected zone) was done first starting from the outside and working inwards. This was followed by vaccination in the second (buffer zone) and third zone (Northern part of country). In 2017, re-vaccination of all susceptible animals was performed with BOVIVAX LSD-N Neethling vaccine (M.C.I. Sante Animale, Morocco) without zoning. Together with vaccination total stamping out was implemented in non-vaccinated herds when LSD was diagnosed, modified stamping out of only the infected animals with clinical signs and after laboratory confirmation of a field virus strain was performed when LSD occurred in vaccinated farms [10]. During the LSD epidemic in Serbia in 2016, 225 outbreaks with 267 affected animals were reported [15].

Annual re-vaccination and vaccination of calves derived from vaccinated cattle at the age of 6 months is the vaccination scheme recommended by the vaccine producer. Whilst vaccination will not induce immune response in each vaccinated animal, mass vaccination will provide good overall protection, if more than 80% of the population are vaccinated [12]. A recent immunological study following up LSD vaccination using Neethling, SPP and GTP vaccine strains has shown that vaccination equally stimulates humoral and cell-mediated immunity [16–18]. Although there are studies on the efficiency of vaccination after a single vaccine application, to our knowledge there are no published studies on the humoral response to the LSDV Neethling vaccine in the field after vaccination and re-vaccination.

Humoral immune response can be investigated by using virus neutralization test (VNT), indirect fluorescent antibody test (IFAT) and ELISA [1, 19]. So far, VNT is the only serological test validated by the OIE with a high specificity for detecting capripoxvirus-specific antibodies [1]. In serological investigations, IFAT should be used with caution, considering the described cross-reactivity with bovine papular stomatitis virus and other poxviruses [1]. A recently developed double antigen ELISA from ID vet® with a high specificity and sensitivity for capripoxvirus antibody detection according to the manufacturer, could fill the gap of serological tools for mass screening.

The aim of this longitudinal study was to investigate the humoral immune response of vaccinated cattle during the re-vaccination campaign as well as of newborn calves after colostrum intake. In addition, a comparison of the results obtained by standard serological methods (VNT and IFAT) with the results obtained by using a commercially available ELISA in order to prove suitability of ELISA was performed.

## Results

### Sampling and on-farm examination

Seventy-nine cattle from twenty farms vaccinated in 2016 and re-vaccinated in 2017 were included in the study. Of all tested animals, two young heifers were not vaccinated in 2016, but all seventy-nine animals were vaccinated in 2017. Vaccination of all cattle in 2016 and 2017 was done by the same veterinary service. Except for one farm, where cattle were kept on pasture and indoors, all animals were housed indoors only with approximately 200–500 m distance between farms without mixing of herds. On the vaccination day and during the study all cattle were healthy with no clinical signs of LSD or any other disease. Swelling at the injection site, which was described by the manufacturer (M.C.I. Sante Animale, Morocco) as a side effect of vaccination in vaccinated cattle, did not occur in this study. At the last time point of sampling five animals were excluded from the investigation because the owners had sold them for slaughter with no clinical signs of disease or other health problems. Passive immunity investigation was performed with 17 re-vaccinated cows and 20 calves (three cows gave birth to twins).

### VNT

In VNT 27 cattle were tested positive after the first sampling, the highest antibody titer was 1:512. A secondary response was seen 1 month after re-vaccination with an increase in the number of seropositive animals up to 60 together with an increase of antibody titers (Table 1). Compared to the results before re-vaccination 5 animals had a higher antibody titer than 1 month after re-vaccination (320–256; 512–320; 256–200; 128–64 and 100–80). The number of seropositive animals decreased from 60 to 42 five months after re-vaccination together with the antibody titer. Higher antibody titers were observed in 7 animals 5 months after re-vaccination compared to the results from 1 month after re-vaccination (320–640; 256–512; 100–128; 50–800; 256–320; 80–128 and 25–40).

**Table 1:** Number of animals listed by the titer of LSDV specific antibody detected with VNT from three sampling time points

Number of animals by time of sampling			
Antibody titer	before re-vaccination	1 month post re-vaccination	5 months post re-vaccination
over 300	3	6	6
200–300	3	10	2
100–200	8	14	9
50–100	5	13	7
10–50	8	17	18
under 10 (negative)	52	19	32

### Ifat

Analyzing samples from before re-vaccination, 26 cattle were seropositive with antibody titers from 40 to more than 160. A secondary response was observed 1 month after re-vaccination in 68 positive animals, with 37 tested animals showing an antibody titer of 160. A drop in the number of seropositive animals from 68 to 43 was seen 5 months post re-vaccination, with 19 animals showing antibody titers of more than 160 (Table 2). Sensitivity (Se) and specificity (Sp) were calculated with results from VNT showing Se_IFAT_ 85% and Sp_IFAT_ 86% with 0.90 positive predictive values (ppv) and 0.80 negative predictive values (npv).

**Table 2:** Number of animals listed by the titer of LSDV specific antibody detected with IFAT from three sampling time points

Number of animals by time of sampling			
Antibody titer	before re-vaccination	1 month post re-vaccination	5 months post re-vaccination
over 160	10	37	19
80	3	22	13
40	13	9	11
border line	7	8	9
under 40 (negative)	46	3	22

### Elisa

**Fig. 1:**
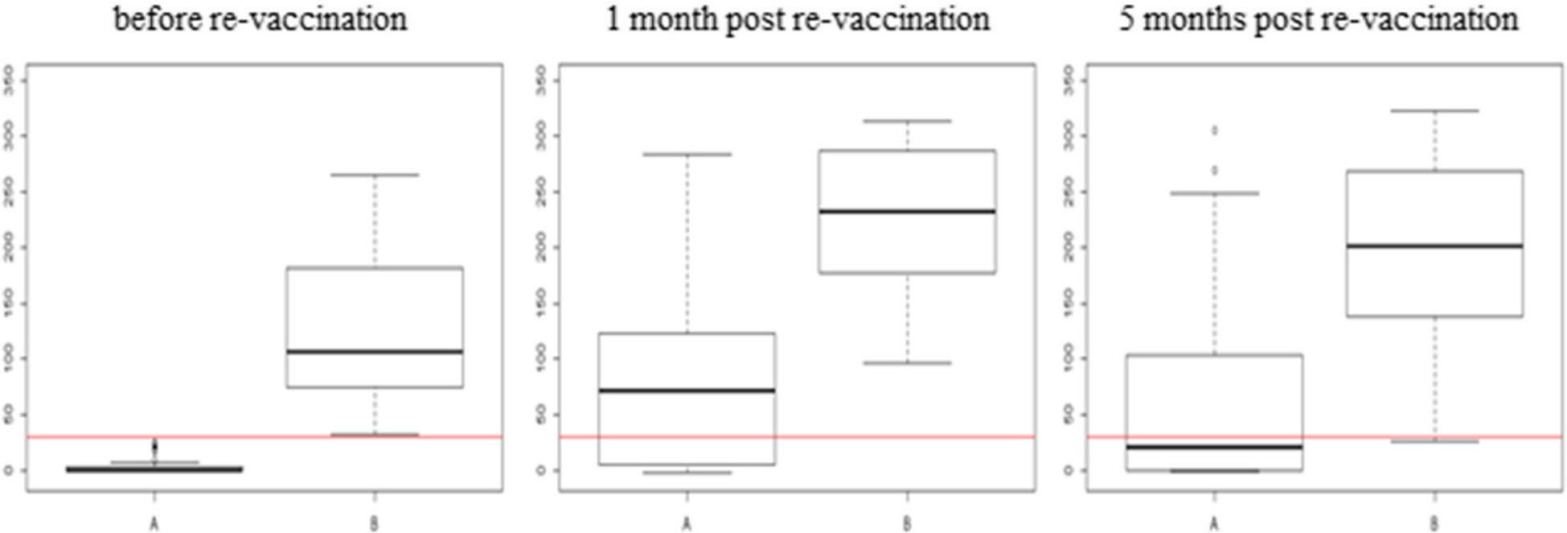
Immune response detected with ELISA from three sampling time points. **a** – non-responders after first sampling; **b** – responders after first sampling; red line – cut off value ≥30

At the first time point of sampling 26 cattle were positive in the ELISA. Like in IFAT and VNT an increase in the number of seropositive animals to up to 58 was observed 1 month after re-vaccination with an increase of S/P % (percentage of positivity compared to the positive control). Five months after re-vaccination the number of seropositive animals dropped to 43 together with a drop of S/P % except in 7 animals (234–268; 116–306; 165–233; 178–318; 258–270; 306–323 and 20–117) which had higher S/P % compared to 1 month after re-vaccination (Fig. 1). Higher sensitivity and specificity were observed with VNT (91 and 87%, respectively, with 0.89 ppv and 0.88 npv) compared to IFAT (Se_ELISA_ 88% and Sp_ELISA_ 76%, with 0.82 ppv and 0.84 npv).

### Comparative serological analysis

**Fig. 2:**
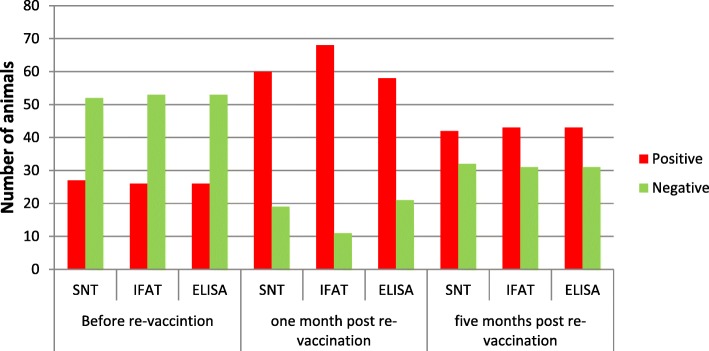
Graphic view of detected positive and negative cattle with three tests from three sampling times showing secondary response

Detection of LSD-specific antibodies was possible at all three time points of sampling and with all three tests used (Fig. 2). A secondary response was observed 1 month after re-vaccination with a significant increase in seropositive animals compared to the results before re-vaccination in all three tests. Even though a secondary response was achieved, there were still some cattle which did not seroconvert after re-vaccination (VNT 19; IFAT 10 and ELISA 21) (Table 3). A decrease in the number of seropositive animals from 1 month to 5 months after re-vaccination was observed with all three tests. With all applied serological methods, at 1 and 5 months post re-vaccination the number of animals testing positive was significantly higher than before re-vaccination (Table 4).

**Table 3:** Detection of positive and negative cattle by sampling time point showing secondary response after re-vaccination

		before/1 m. p. re-vaccination	before/5 m. p. re-vaccination	1 / 5 m. p. re-vaccination
VNT	positive/stay positive	27	23	42
	positive/turned negative	0	2	15
	negative/turned positive	33	19	0
	negative/stay negative	19	30	17
IFAT	positive/stay positive	25	20	42
	positive/turned negative	1	4	22
	negative/turned positive	43	23	1
	negative/stay negative	10	27	9
ELISA	positive/stay positive	26	22	42
	positive/turned negative	0	1	13
	negative/turned positive	32	21	1
	negative/stay negative	21	30	18

**Table 4:** Statistical significance calculated between tests and between sampling time points

Time of sampling	Test	*P* value	Result
before re-vaccination	VNT/IFAT	1	*p* > 0.05
	VNT/ELISA	1	*p* > 0.05
	IFAT/ELISA	1	*p* > 0.05
1 month post re-vaccination	VNT/IFAT	0.1549	*p* > 0.05
	VNT/ELISA	0.855	*p* > 0.05
	IFAT/ELISA	0.0738	*p* > 0.05
5 months post re-vaccination	VNT/IFAT	1	*p* > 0.05
	VNT/ELISA	1	*p* > 0.05
	IFAT/ELISA	1	*p* > 0.05
before / 1 month post re-vaccination	VNT/VNT	<0.0001	*p*<0.0001
before / 5 months post re-vaccination	VNT/VNT	0.0059	*p*<0.01
1 month / 5 months post re-vaccination	VNT/VNT	0.0161	*p*<0.05
before / 1 month post re-vaccination	IFAT/IFAT	<0.0001	*p*<0.0001
before / 5 months post re-vaccination	IFAT/IFAT	0.0021	*p*<0.01
1 month / 5 months post re-vaccination	IFAT/IFAT	0.0001	*p*<0.001
before / 1 month post re-vaccination	ELISA/ELISA	<0.0001	*p*<0.0001
before / 5 months post re-vaccination	ELISA/ELISA	0.0021	*p*<0.01
1 month / 5 months post re-vaccination	ELISA/ELISA	0.0601	*p* > 0.05

### Maternally derived antibodies

The results of the investigation of maternally derived antibodies are presented in detail in Table 5. Only 2 of the 17 cows reacted negative in less specific IFAT. For 9 of the 17 cows a positive VNT titer could be defined. Seven of these 9 VNT-positive cows reacted positive also in the ELISA. The analyses of the pre-colostral collected serum sample showed clear negative results in ELISA and IFAT. Passive antibody transfer from vaccinated cows to newborn calves was observed at 14 days after birth. All 9 VNT-positive calves reacted also positive in the ELISA and all 11 VNT-negative calves showed also a negative result in the ELISA. Discrepancies for the detection of LSDV specific antibodies between cows and newborn calves at the age of 14 days were observed in VNT and IFAT (ID #11, #14 and #15), but not in ELISA.

**Table 5:** Detailed results of passive LSDV specific antibody transfer from vaccinated cows to new-borne calves

Cow				Calf					
ID	Serum			ID	0 day old		14 days old		
	ELISA	VNT	IFAT		ELISA	IFAT	ELISA	VNT	IFAT
1	−2	<1:10	1:80	1	−2	<1:40	-1	<1:10	1:80
2	-1	<1:10	1:40	2	−2	<1:40	0	<1:10	1:160
3	−2	<1:10	1:80	3	−2	<1:40	0	<1:10	1:40
4	-2	<1:10	1:160	4	-2	<1:40	−1	<1:10	1:160
5	49	1:32	1:160	5	−1	<1:40	101	1:32	1:80
6	383	1:256	1:160	6	−1	<1:40	305	1:200	1:160
7	140	1:100	1:160	7	−2	<1:40	227	1:64	1:80
8	−2	<1:10	1:160	8	−2	<1:40	−1	<1:10	1:40
9	−3	<1:10	<1:40	9	−2	<1:40	−2	<1:10	<1:40
10	241	1:128	1:160	10	−2	<1:40	315	1:200	1:160
11	−3	1:16	1:80	11	−1	<1:40	−3	<1:10	1:40
12	281	1:128	1:160	12	−1	<1:40	269	1:80	1:160
13	68	1:20	1:80	13	−2	<1:40	71	1:40	1:80
				13	−1	<1:40	65	1:20	1:40
14	1	<1:10	1:160	14	−2	<1:40	−2	n.d.	<1:40
				14	−2	<1:40	−1	<1:10	<1:40
15	3	1:25	1:160	15	−2	<1:40	1	n.d.	<1:40
16	190	1:128	1:80	16	−2	<1:40	192	1:64	1:80
				16	−3	<1:40	254	1:80	1:80
17	−3	<1:10	<1:40	17	−3	<1:40	−3	<1:10	1:80

Cut off values: ELISA ≥30; VNT ≥1:10 and IFAT ≥1:40. n.d. not determined

### PCR

EDTA blood, nasal and mouth swabs from all tested animal reacted negative in the PCR showing no presence of LSDV field strain or Neethling vaccine strain genome. All internal process, negative and positive controls reacted as expected (data not presented).

## Discussion

In the presented study we investigated the development of the humoral immune response against LSDV during the process of re-vaccination in cattle over a time span of 5 months, additionally comparing the performance of different serological techniques. Samples for the study were collected in an area in Serbia without reports of previous LSD outbreaks. Furthermore, all clinical investigations during the different sample collections as well as negative testing of all collected samples by capripoxvirus-qPCR confirmed freedom of LSDV field strain in the analyzed farms.

Besides the important cell-mediated immune response, the easier to detect humoral immune response can provide significant information about the success of vaccination, but is time limited. Even though no new LSD outbreak was reported in Serbia after implementation of a blanket vaccination in the cattle population in 2016, the status of immunity against LSDV was poorly understood. The current knowledge on the time span of antibody detection after vaccination is fairly heterogeneous. A significant increase of capripoxvirus-specific antibody titers is described to be seen from day 21 up to day 42 after vaccination, and antibodies remain detectable for about 7 months [1]. A recent immunological study conducted in cattle after vaccination with LSDV showed that detection of specific antibodies is limited to 40 weeks post vaccination [16]. In the presented study, detection of LSD-specific antibodies in cattle vaccinated in 2016 was possible 46 to 47 weeks after vaccination (35.06% by VNT and in 33.77% by IFAT and ELISA). These results suggest that LSD-specific antibodies might be detected longer than described so far. In our study, a secondary response was observed after re-vaccination showing a significant increase in seropositive animals in all three tests used. The detection of rising antibody titers in some animals 1 month after re-vaccination is concurrent with previously described findings [16]. Higher level of detected antibodies can be attributed to better immune response to vaccination. In the study of Kitching [20] it was demonstrated that serum from sheep immune to capripoxvirus infection protected recipient sheep against challenge with virulent capripoxvirus confirming that antibody are sufficient in protection against capripoxvirus infection. Nevertheless, a robust number of cattle remained serologically negative after re-vaccination. Missing seroconversion in a certain number of animals vaccinated against LSD was already described by poor immunogenicity due to over-attenuation of Neethling and KSGP vaccine [14]. In the same study of Gari et al. [14] animals which were vaccinated with Gorgon vaccine, only 50% developed immune response after vaccination, but all animals were protected against capripoxvirus challenge. All three tests target the same biological factor and no significant statistical difference was seen between them. A mismatch of detected positive and negative cattle was seen to occur between ELISA and VNT in 26 cases, between ELISA and IFAT in 40 cases and between VNT and IFAT in 34 cases. In the study of Babiuk et al. [21], the same nonconformity of serological tests was reported, which was explained by detection of different anti-capripoxvirus antibodies with the different tests used.

Use of standard serological tests (VNT and IFAT) for investigation of the humoral immune response has its disadvantages, as they are time consuming, require handling of live virus, and high-throughput analyses are not possible. VNT as the only validated test was used as gold standard for confirmation of the results obtained with two other tests. The main disadvantage of using VNT is that it requires live LSDV which is restricted to be used in national reference laboratories operating in high-level bio-containment facilities [22].

Analysis of the results of three serological tests used in this study shows that IFAT gave the highest number of seropositive animals 1 month after re-vaccination followed by VNT and ELISA. The high sensitivity of IFAT could be attributed to cross-reactivity of IFAT with bovine papular stomatitis virus or with pseudo-cowpox virus observed at a lower dilution (≤1/8), [1, 23]. It must be noted though that there was no documented clinical infection with bovine papular stomatitis virus or pseudo-cowpox virus in animals included in this study and that by starting dilution at 1:40 we aimed to avoid a false positive reaction. In the study of Gari et al. [19] Se and Sp of IFAT were higher, 92 and 88%, respectively, compared to our study, where Se_IFAT_ was 85% and Sp_IFAT_ 86%.

The newly developed ELISA capripoxvirus double antigen multi-species, with a claimed specificity of > 99.7% by the manufacturer shows suitability for serological investigation of a vaccinated cattle population in the field with nearly the same results as VNT. Calculating sensitivity and specificity of ELISA was estimated to be Se_ELISA_ 91% and Sp_ELISA_ 87% to VNT and Se_ELISA_ 88% and Sp_ELISA_ 76% to IFAT. The ELISA test has many advantages compared to standard serological tests making it ideal for time- and cost-efficient analysis of a large number of samples in mass screening activities.

The component of our study looking into the passive transfer of antibodies confirmed that seropositive cows provide colostral antibodies to their calves. For detection of passive antibody transfer IFAT again shows the highest number of positive animals compared to VNT and ELISA. In general, suitability of the ELISA for investigation of passive antibody transfer was shown by the fact that failure of detection of LSDV-specific antibodies in calves at the age of 14 days did not occur. Nevertheless, the missing of LSDV-specific antibodies in new-borne calves at the age of 14 days despite the detection of antibodies in the according mothers by VNT and IFAT (ID #11, #14 and #15) could be explained by the fact that not all cows will provide the same colostrum quality [24]. On the other hand, one calf (#17) was detected positive at the age of 14 days only with IFAT although the mother was negative. This finding can be attributed to a higher concentration of antibodies in colostrum than in the serum of cow [25] and to the fact that with different tests different anti-capripoxvirus antibodies could be detected [21]. For successful transfer of antibodies via colostrum four factors must be respected: feeding colostrum with high immunoglobulin concentration (> 50 mg/mL of IgG), feeding an adequate volume of colostrum, feeding colostrum promptly after birth, and avoiding bacterial contamination of colostrum [26–28]. In this study, all calves were in good condition at birth and took colostrum in sufficient amounts between the first 30 min to 2 h after birth, with no clinical signs of disease during the first 14 days of life.

According to the vaccine producer, vaccination of calves derived from vaccinated mothers should be done at the age of 6 months because of maternally derived antibodies. From recent studies of Agianniotaki et al. [29] maternally derived antibodies can be detected 3 days after feeding with colostrum until 3 months of age, leaving a potential vaccination gap of 3 months in some calves.

## Conclusion

Based on the results of this study, it can be concluded that re-vaccination leads to a secondary response in the cattle population and significantly increases the number of animals with detectable antibody titers. The detection of capripoxvirus-specific antibodies nearly eleven months post vaccination (46 to 47 weeks) in a certain number of animals with all three tests shows that in individual cattle humoral immune response can last longer than 7 months. The commercially available capripoxvirus ELISA delivered very good results compared to VNT or IFAT. The ELISA will be the most useful serological technique for high throughput analyses.

## Methods

### Sample collection

Seventy-nine animals from 20 randomly selected farms in Vrdila village in Kraljevo municipality with no previously detected LSD outbreaks in this area were included in the longitudinal serological investigation. Sample collection was performed three times in 2017. The first sample collection was performed before re-vaccination in 2017, the second 1 month after re-vaccination in 2017 and the third 5 months after re-vaccination in 2017. Nasal and oral swabs were collected at all three time points of sampling for detection of a potential circulation of Neethling vaccine strain and field strain of LSDV.

During sample collections, a clinical evaluation of all animals was performed looking for unspecific clinical signs of disease such as fever, mastitis, nasal, oral or ocular discharge and presence of visible or palpable nodules. For the investigation of passive immunity transfer in new-born calves, 17 cattle from two farms with good calving management and strong biosecurity measures were selected. All calves were separated from their mothers after birth. The newborns were fed with 2 l of colostrum between 30 min and 2 h after birth. Plain vacutainer tubes without anticoagulant BD Vacutainer^tm^ 4,055,269 (Belliver Industrial Estate, UK) and with EDTA anticoagulant Vacuette® tube A170434K (Greiner Bio-One, Austria) were used for blood collection from all cattle via coccygeal venipuncture and from calves via Jugular venipuncture. Blood from cows was collected on the day of calving and from calves before taking colostrum and at the age of 14 days. After sample collection all animals were further used in agriculture production.

### Sample processing

Blood samples without anticoagulant were allowed to clot for 3 h at room temperature and then serum was extracted by centrifuging at 2000 RPM for 20 min and aliquot in 1.5 ml centrifugal tubes. Swab material was collected using synthetic swabs (Copan, Italy) and immediately immersed into the Dulbecco’s Modified Eagle’s Medium (DMEM; Gibco, USA) supplemented with 1% of antibiotics (Penicillin 1000 IU - Streptomycin 10 mg; Sigma, Germany) and 1% of antimycotic (Amphotericin B; Sigma, Germany). All samples were stored at − 20 °C until examination.

### Serological methods

Detection of LSDV specific antibodies from investigated animals was assessed by using virus neutralization test (VNT), indirect fluorescent antibody test (IFAT), and ELISA (ID vet® Capripox Double Antigen Multi-species, Montpellier, France). VNT is the only OIE-validated test for detection of LSDV specific antibody. For VNT and IFAT, MDBK cells were grown using DMEM supplemented with 10% foetal calf serum (FCS) and incubated at 37 °C with 5% CO2.

### VNT

All tested serum samples including positive and negative control serum were incubated at 56 °C for 30 min. To assess the titer of LSDV specific antibody, triplicates of tested serial 2-fold dilution series were titrated against a constant titer of Neethling vaccine strain 100TCID_50_ (The Pirbright Institute, Pirbright, UK)_._ The tested serum, positive and negative control serum were diluted in DMEM without FCS (from 1:10 to 1:1280). Serum dilutions and the fixed amount of virus strain were incubated for 2 h at 37 °C. After the neutralization step 100 μL suspension of MDBK cells in DMEM with 10% FCS was added to each well. In each test one control plate was included with only virus titration and titration of positive and negative control serum. Plates were incubated at 37 °C, 5% CO2. After 4 days plates were examined for appearance of cytopathogenic effect (CPE) and final reading was taken on day 7. Results were recorded and titer was calculated using the Spearman and Kaerber method [30]. Samples with an antibody titer of ≥1:10 were considered as positive.

### Ifat

MDBK cells were incubated for 1 day in 96 well cell culture plates to obtain an 80–90% confluent monolayer. Cells were infected with 100 μL of 100TCID_50_ virus suspension of Neethling vaccine virus strain (The Pirbright Institute, Pirbright, UK). Rows one, five, and nine were not infected for surveillance of non-specific interaction of tested sera and cells. After 48 h of incubation at 37 °C and 5% CO2 infected and non-infected MDBK cells were fixated with 200 μL Acetone-Methanol 1:1 for 20 min. After fixation, MDBK cells were blocked adding 100 μL Saponin-Blocking-Buffer (0.2% *w*/*v* BSA, 0.1% w/v NaN_3,_ and 0.05% Saponin) for 30 min to reduce non-specific reaction. Tested serum, positive and negative control serum were prepared in log dilutions 1:40, 1:80 and 1:160 in Saponin-Blocking-Buffer. Anti-bovine gamma globulin conjugated with fluorescein isothiocyanate obtained from rabbit was prepared at a dilution of 1:400 in Saponin-Blocking-Buffer and 100 μL were added to each well. Reading of plates was done using Carl Zeiss Florescence microscope under 40X magnification. The test was considered valid if positive sera gave clear green fluorescence at each dilution step and negative serum was without florescence signal. Tested sera were considered positive if at a lower dilution clear green fluorescence signal was present and negative when no fluorescence signal was seen.

### Elisa

Antibody detection via ELISA was performed using ID Screen® Capripox double antigen Multi-species ELISA kit from ID vet® (Montpellier, France) according the manufacturer’s instructions.

### Polymerase chain reaction

DNA from swab samples and EDTA blood together with internal control RNA (IC-RNA) added to each sample [31] was extracted using the NucleoMag Vet Kit (Macherey-Nagel GmbH, Germany) with King Fisher Flex (ThermoFisher Scientific, Finland) machine. Detection of viral genome from material was done by pan real-time PCR reaction based on P32 capripoxvirus gene amplification using the previously published protocol of Bowden et al. [32] modified according to Dietze et al. [33] and with included IC system [31].

### Statistics

Statistical analysis was performed by Fisher exact test with program GraphPad Prisma version 7 (San Diego, CA, USA). Statistical significance was calculated between tests from each sampling time point and between the same test from different sampling time points. Calculation of sensitivity and specificity of the tests was performed with cattle from all three sampling time points.

## Abbreviations

CPE: Cytopathogenic effect
GTP: Goat poxvirus
IFAT: Immunoflurescent antibody test
LSD: Lumpy skin disease
LSDV: Lumpy skin disease virus
npv: Predictive negative value
ppv: Predictive positive value
S/P %: Percentage of positivity compare to the positive control
Se: Sensitivity
Sp: Specificity
SPP: Sheep poxvirus
VNT: Virus neutralization test
